# Klein and Loftus's model of trait self-knowledge: the importance of familiarizing oneself with the foundational research prior to reading about its neuropsychological applications

**DOI:** 10.3389/fnhum.2013.00699

**Published:** 2013-10-21

**Authors:** Stanley B. Klein

**Affiliations:** Department of Psychological and Brain Sciences, University of CaliforniaSanta Barbara, CA, USA

**Keywords:** self, memory, trait-knowledge, neuroscience, theory, clinical populations, model testing

This article has two goals. First, I want to respond to concerns voiced by Ruby (2013). Since these concerns are not limited to Ruby, they provide an opportunity to address the issues she raises for a broad audience of neuroscientists. Second, and related to my first goal, I want to alert investigators who are familiar only with our neuropsychological investigations of self-knowledge to our earlier work on model construction. A familiarity with this foundational research can help avert concerns and issues likely to arise if one is aware only of neuropsychological extensions of our work.

## Ruby's concerns

In a recent article in this journal, Ruby (2013) proposed that cognitive neuroscience may provide theoretical grounding for many tenets of Psychoanalytic Theory. For example, neuroscience may help situate unconscious memories of experienced events (UMEEs)—which play a critical role in psychoanalysis—in a more scientifically respectable context.

I have no wish to debate the merit of Ruby's thesis. I do, however, take issue with some of the “evidence” she marshals in its support. Specifically, a considerable proportion of her paper laments the lack of attention she feels has been accorded to UMEEs in the investigations of trait self-knowledge. This negative assessment apparently was motivated by her reading of Klein and Gangi (2010), a review paper describing our attempts to extend our work on trait self-knowledge to individuals suffering clinical dysfunction (e.g., amnesia, autism, dementia). I quote the relevant sections of her paper:

“The consideration of UMEE has been lacking for example in research investigating memory and the self, as illustrated by the article of Klein and Gangi (2010)… the authors aimed to better understand the link between the different types of self-related memory systems, by investigating the representation of self-personality traits in patients with amnesia. They report results showing that some patients with episodic amnesia … update the representation of their own personality traits. For Klein and Gangi, these cases showed that episodic and semantic memory systems were separate and independent. Interestingly, they also considered … the possibility that UMEE may participate in the updating of personality traits since they wrote “K.C. not only had access to semantic knowledge of his own personality traits, but he was also able to acquire new knowledge about his personality. Yet this updating occurred without his being able to episodically recollect any information about the behavioral events on which this updating presumably was based.” Unfortunately, the authors did not develop this point and did not discuss the hypothesis of the updating of personality traits based on the UMEE … However, (their findings) do not exclude the possibility that UMEE participate in the formation of semantic knowledge” (p. 2).

I believe Professor Ruby's concerns can be addressed by reference to the body of work that served as the conceptual basis for our later neuropsychological investigations. Our argument that episodic and semantic memory systems are (functionally) independent was not based on data from our case studies. Rather it was based on empirical work conducted with mentally healthy participants; only later did we apply these findings and the model they resulted in to patients suffering cognitive impairments.

## A model of trait self-knowledge in non-clinical populations

The goal of our formative work was to determine whether trait-relevant behavioral exemplars play a role in trait judgments about the self. To address this question, we initiated a program of research using cognitively and neurologically healthy individuals. Our patient studies came later, after we already had formulated and tested our model of trait self-knowledge. The investigations of individuals with well-circumscribed cognitive and neurological impairments seemed a natural way to extend our initial findings and refine our model.

Our non-clinical studies used a variety of methodologies, procedures and contextual factors (e.g., self-at-home, self-at-school) to examine the two most widely adopted models of the mental representation of trait self-knowledge: The *computational* model (i.e., trait judgments are based on access to behavioral exemplars in memory) and the *abstraction* model (i.e., trait judgments are based on access to pre-computed semantic summaries). Each model had advocates, but deciding between them had proven difficult. Our strategy was to adopt experimental methods that would enable us to pit these models against one another, thereby allowing us to assess their respective merits.

Due to “length of text” constraints, I focus on the results of our priming studies. However, the reader should be aware that to garner convergent support we employed a variety of methods–e.g., transfer appropriate processing in recognition memory; Dunn and Kirsner's (1988) technique of reversed association [reviewed in Klein et al. (2008); Klein and Lax (2010)].

According to the logic of our priming methodology, if trait judgments require the activation of trait-relevant behavioral exemplars in memory (regardless of whether or not that activation results in conscious awareness–see below), two things should happen: (1) an initial request to judge whether a trait is self-relevant should prime (i.e., speed up) the subsequent performance of a task requiring participants to retrieve trait-relevant exemplars from memory (since exemplars already will have been activated by the judgment task), and (2) a request to retrieve trait-relevant exemplars from memory should prime performance of a subsequent trait judgment task, since the retrieval task will have activated exemplars required to make a trait judgment.

By contrast, if trait judgments are based on semantic abstractions rather than behavioral exemplars, then the activation of exemplars will not play a part in the judgment process. Accordingly, exemplar-based priming should not be observed.

To cut to the chase, in over a dozen studies we consistently found that–except in certain, theoretically predicted circumstances—priming did not occur. We concluded that trait judgments typically are not based on either the conscious or unconscious activation of behavioral exemplars; rather—in accord with the trait abstraction model—they appear to depend on access to pre-computed trait summaries in semantic memory.

As noted, priming was observed in certain situations. However, these “exceptions to the rule” were predicted by the abstraction model. Equally important, these “exceptions” demonstrated that our priming methodology was sufficiently sensitive to detect trait-relevant behavioral exemplars–whether conscious or not–when exemplars were predicted, on the basis of theory, to play a part in the judgment process (for a review see Klein et al., 2008).

With model in hand, we turned our attention to patients suffering from neuropsychological disorders to see whether our theory could account for—and perhaps provide insight into—impairments of self-knowledge exhibited by individuals suffering cognitive dysfunction (e.g., Klein and Gangi, 2010).

## Questions concerning our neuropsychological findings likely to arise from a lack of familiarity with the foundational research

Since, with one exception, all of our non-clinical research on self-knowledge was published in social psychology journals, many neuroscientists may not be aware of the work that provided the empirical and theoretical foundation for the model we subsequently applied to clinical populations. Our neuropsychological investigations of self-knowledge recently have captured the interests of the neuroscience community (e.g., Renoult et al., 2012; Martinelli et al., 2013; Picard et al., 2013; Prebble et al., 2013); therefore, it seems a good time to direct attention to that earlier body of work [a comprehensive summary can be found in Klein et al. (2008)]. Familiarity with this formative literature may help to preempt misunderstandings and address questions likely to arise when one's acquaintance with our model of self is based primarily on what can be gleaned from its neuropsychological applications.

For example, an appreciation of this research reveals that we were very concerned about the potential effects of UMEEs on trait judgments. Accordingly, we employed a variety of tasks that had been shown not to require conscious access to the content assumed to mediate their successful performance. While the findings thus far mentioned do not directly address the possibility that UMEEs play a role in the formation of semantic summaries, data presented in a later section of this article casts some doubt on that hypothesis as well.

Our interest in the potential effects of unconscious behavioral exemplars on the trait judgment process is put in sharp relief by Klein et al. (1992). While explaining the logic of priming, we noted that this technique “does not require that behavioral exemplars be consciously retrieved to exert an influence on subsequent task performance. On the contrary, we share with many exemplar theorists the view that the effects of individual exemplars often will be outside of conscious awareness … We argue only that if in the process of performing an initial task, behavioral memories are activated, these memories will be more accessible for subsequent retrieval in a trait judgment task than had they not been activated.” (p. 743).

As noted, our research yielded a patchwork of positive and null priming results (e.g., Klein et al., 2008). Accounting for these findings by appeal to UMEEs would seem a daunting undertaking. A UMEE account would have to explain “when and why” priming occurred and “when and why” it did not. It would have to accommodate a complex pattern of findings, obtained with a broad range of experimental conditions (including variations in method, population, context and experience). Even if one held that UMEEs play a role only in the formation of semantic summaries, one still would need to explain “when and why” those summaries are formed–and thus, available for trait judgments–in some situations but not in others. Lacking a principled basis for predicting the full pattern of our results, an advocate of UMEE theory would have to remain open to the possibility that a unique explanation might be required for a number of our “seemingly” conflicting findings.

By contrast, what appears to be an erratic collection of outcomes can readily be transformed into an orderly, predictable and programmatic set of findings by appeal to the abstraction model conjoined with just two additional, theoretically-motivated principles—i.e., episodic and semantic memory are functionally independent; the formation of trait-summaries varies as a function of trait-relevant behavioral experience. Of course, Occam's razor is a guiding principle, not a metaphysical mandate. But, assuming that parsimony is a desirable heuristic in theory construction, it is hard to see how a model based on UMEEs can accommodate our complete set of findings in as economical a manner as the abstraction model.

In the text quoted above, Ruby raises a second concern. How, she asks, can amnesic patient K.C. update his trait self-knowledge when he is unable to recollect any information about the behavioral events on which this updating presumably is based? The most likely answer, in her opinion, is that updating his semantic trait-knowledge results from the activation of trait-relevant UMEEs. “How,” she wonders, “could one otherwise explain (these) results?” (p.2; parenthesis added for clarification).

It turns out that our early work speaks to Ruby's puzzlement. Sherman and Klein (1994) demonstrated that trait updating occurs by processes occurring at encoding—not by the activation (conscious or unconscious) of experiences already in memory. Indeed, since in this and other studies, we presented the trait-relevant behavioral information on which summaries presumably were based on-line via computer, there was little opportunity to access pre-stored UMEEs in memory^1^.

These findings fit nicely with Tulving's (1995) SPI model of memory, according to which, information is stored in semantic memory during initial processing—not as the result of some serial process in which content first is activated from episodic memory and then converted to semantic knowledge.

Thus, empirical and theoretical answers to Ruby's query (i.e., “how can our updating results be explained without recourse to UMEEs?”) already exist. But one needs to know where to look. The purpose of this paper is to provide that guidance.

Further complicating matters for a UMEE model of the updating process, Klein and Gangi (2010) also reported the case of K.R., a patient with Alzheimer's Dementia. In addition to seriously compromised episodic memory, K.R. showed severe impairment in her ability to add knowledge to semantic memory (as often is the case in advanced stages of dementia; e.g., Westmacott et al., 2004). And,— as predicted by the abstraction model—absent the ability to update semantic memory, K.R. should be incapable of updating her trait self-knowledge to accommodate changes in her personality wrought by dementia. This is exactly what we (and others) found (e.g., Klein et al., 2003).

By contrast, K.C.'s semantic memory is essentially uncompromised (e.g., Tulving et al., 1991; Tulving, 1993). Accordingly, his trait updating abilities–as predicted by our model–remain largely intact despite severe episodic amnesia. Considering all of the evidence (patients' semantic memory status, theoretical modeling, and non-patient empirical work), the differences we observed in patients' ability to update trait self-knowledge seem most parsimoniously attributable to differences in the status of their semantic memory–not to differential access to UMEEs.

Non-the-less, the empirical and theoretical considerations presented cannot *conclusively* rule out a role for UMMEs in the formation of the semantic trait summaries that are stored and later accessed during the trait judgment process. The possibility that the formation of the semantic knowledge on which trait judgments subsequently are based may be due, in part, to unconscious mental activity is something which currently available methodologies cannot definitively address.

## Footnotes

^1^These studies examined the formation and representation of trait knowledge about an artificial “other”. However, our work has shown that the same processes operate whether the target is “self” or “other.”
